# Alliance-based modeling of interbank lending networks: insights into risk contagion dynamics

**DOI:** 10.1371/journal.pone.0324981

**Published:** 2025-06-05

**Authors:** Ziwen Jiang, Xu Jiang, Huanlan Yan, Yu Xie, Jiamin Yao

**Affiliations:** 1 XuBeihong Art Academy, Shanghai Maritime University, Shanghai, China; 2 Academy of Arts & Design, Tsinghua University, Beijing, China; 3 College of Computer Science and Technology, Tongji University, Shanghai, China; 4 College of Information Engineering, Shanghai Maritime University, Shanghai, China; Central Bank of Brazil, BRAZIL

## Abstract

The financial market, a highly intricate and dynamic system, relies heavily on banks as its central pillars. The interactions among these banks are pivotal in shaping the stability and efficiency of the overall financial system. A comprehensive understanding of the interbank relationships and network structures is essential to uncover the underlying mechanisms that drive financial markets. This paper presents a novel approach to modeling interbank lending as a dynamic network, aiming to explore the influence of banking alliances on risk contagion dynamics. While extensive research has been dedicated to interbank lending strategies, the role of long-term cooperative alliances and their structural effects on lending behavior has remained largely underexplored. In this study, we propose an innovative interbank lending strategy that integrates alliance relationships, facilitating the construction of networks that more accurately represent the characteristics of real-world banking alliances. Through a series of simulation experiments, we investigate the risk contagion dynamics within these alliance-based networks under different external shock scenarios. Our findings provide valuable insights into the resilience and contagion mechanisms of banking alliance networks, offering practical recommendations for both financial institutions and regulators in designing more effective risk mitigation strategies.

## Introduction

In modern financial systems, the intricate interconnectedness of banks forms a complex web of relationships that is fundamental to both market stability and efficiency. These connections, often manifested through interbank lending and borrowing activities, constitute dynamic networks where the behavior of individual banks can lead to far-reaching systemic effects [1]. While this interconnectedness promotes liquidity and facilitates capital flow, it simultaneously introduces significant vulnerabilities. A disturbance in one bank can trigger a cascading effect throughout the network, amplifying risks and threatening the stability of the entire financial system [2]. Consequently, understanding the structural relationships and the dynamic interactions within these networks is crucial for effectively assessing and mitigating systemic risks[3].

Interbank markets inherently form dynamic networks through lending and borrowing activities, where banks are tightly interconnected [4]. Modeling these networks provides valuable insights into how localized interactions influence the global behavior and overall stability of the financial system [5]. Such models are instrumental in identifying underlying network structures, monitoring the evolution of interbank relationships, and, most importantly, understanding the mechanisms driving risk propagation and contagion within the banking system.

Existing literature on interbank lending networks has predominantly concentrated on factors such as historical lending relationships, counterparty creditworthiness, and asset sizes [6]. While these elements are undeniably important in understanding interbank behaviors, they fail to account for the critical role of long-term cooperative relationships between banks—an essential characteristic of real-world financial systems. In practice, many interbank transactions are built on alliances, which are enduring collaborative relationships rooted in trust, mutual benefit, and shared objectives [7]. These alliances are influenced by both external factors, such as market conditions, regulatory policies, and economic cycles, and internal factors, including risk preferences, information-sharing practices, and interdependence among banks [8]. Particularly during financial distress, alliance structures can significantly impact interbank lending behavior, as banks within the same alliance are more likely to offer mutual support [9]. Despite their considerable influence, alliance structures have been largely overlooked in the existing research.

To address this gap, this paper aims to develop a more realistic framework for analyzing systemic risk in interbank networks by explicitly incorporating alliance structures. Our primary objectives are twofold: first, to introduce a novel algorithm that generates interbank networks based on an alliance-driven lending strategy, capturing the dynamics of trust, cooperation, and mutual dependence inherent in these relationships. Second, to develop a data-driven Agent-Based Model (ABM) that simulates the behavior of individual banks within these generated networks. This ABM, calibrated using balance sheet data on bank characteristics from CSMAR, allows us to investigate how alliance structures influence network dynamics and resilience. By simulating the propagation of risks under external shocks of varying magnitudes within these alliance-based networks, we compare their behavior to traditional network structures lacking alliance considerations, thereby facilitating a more nuanced exploration of risk contagion mechanisms.

The contributions of this paper are threefold:

(1) Proposing an alliance-based interbank lending strategy: This study introduces a novel framework that integrates alliance relationships into interbank lending, generating networks that more closely mirror the trust and cooperative dynamics observed in real-world financial systems.
(2) Simulation of Risk Contagion in Alliance-Based Networks: Through extensive simulation experiments, this paper analyzes the behavior of alliance networks under shocks of different magnitudes, offering insights into their resilience and the key factors that influence contagion dynamics.
(3) Provision of Actionable Insights for Risk Management: The findings of this study provide practical recommendations for policymakers and financial institutions, emphasizing strategies to enhance network resilience and mitigate the systemic impact of financial shocks.

This paper is structured as follows: *Related Work* discusses the relevant work on interbank lending decision-making, the network generation algorithm, and the development of the data-driven agent-based model. *Proposed Method* details the specific methods implemented in this paper, including the initialization of the network and the Alliance-Based Interbank Lend-Borrow Relationship Matching. *Experiments* describes the dataset used for model calibration, the results of the alliance division, and the risk contagion simulation experiments. Finally, *Conclusion* concludes the paper by summarizing the key findings and discussing directions for future research.

## Related work

### Interbank lending decision-making

Interbank lending strategies have been widely examined, with emphasis on factors like historical relationships, counterparty creditworthiness, and asset size. For instance, Liu *et al*. [10] integrated historical lending patterns and asset sizes into their evaluation models to analyze lending decisions. Metawa *et al*. [11] developed optimal credit allocation strategies by considering loan-specific attributes such as duration, size, interest rates, and borrower credit scores. Similarly, Le *et al*. [12] highlighted the significant role of cash flow statements in shaping short- and long-term lending decisions among Vietnamese commercial banks. Schelling *et al*. [13] explored how negative interest rates influence banks’ risk-taking and lending practices, demonstrating that deposit-reliant institutions tend to relax loan terms to offset rising funding costs. Additionally, Sánchez Serrano [14] found that elevated non-performing loan ratios are associated with reduced loan growth rates, reflecting the broader implications of credit quality on lending activity.

While these studies contribute valuable perspectives on interbank lending, they primarily focus on static factors and neglect the dynamic influence of long-term cooperative relationships [15,16], such as strategic alliances. These relationships significantly shape network structures and play a crucial role in risk propagation and contagion, areas that require deeper investigation to fully understand their systemic implications.

### Interbank cooperation and alliances

Recent research has also explored cooperative relationships among banks, emphasizing their role in shaping interbank dynamics. Galal *et al*. [17] proposed a multi-dimensional personalization framework leveraging supervised and unsupervised data mining techniques to enhance CRM performance. Their framework integrates multiple dimensions to improve automation and targeting, supported by a theoretical case study, though it lacks extensive practical validation. Khanizad *et al*. [18] utilized game theory to demonstrate how cooperation among banks reduces operational costs and enhances profitability, showing that alliances consistently outperform individual strategies, even in expanding markets with growing demand. Laux *et al*. [19] analyzed European overnight funding networks and found that forming new cooperative ties leads to lower future interest rates, a finding supported by empirical data.

While these studies underscore the importance of alliances in influencing interbank lending and cooperation, they overlook the role of such relationships in risk propagation and contagion within interbank networks. This gap limits a comprehensive understanding of how cooperative structures impact systemic stability.

### Alliance detection in networks

The confidential nature of interbank transaction data makes direct observation of alliance structures challenging [20]. To address this limitation, researchers have adopted community detection and alliance identification methods from network science [21]. Techniques such as the Girvan-Newman algorithm [22], the Louvain algorithm [23], and modularity-based optimization methods [24] have proven effective in inferring cooperative structures through network properties.

The Girvan-Newman algorithm, designed for smaller networks, detects communities by iteratively removing edges with high betweenness centrality. Its adaptability has led to applications across various fields. For instance, the Revised Girvan-Newman algorithm [22] has been applied to educational and transportation networks, while Huang *et al*. [25] proposed a Self-Contained Girvan-Newman algorithm for hazardous material transportation. Similarly, Jazayeri *et al*. [26] tailored an improved version for water network analysis.

For larger networks, the Louvain algorithm is widely used due to its efficiency and scalability. It identifies communities by optimizing modularity, making it ideal for networks with millions of nodes. Enhancements to the algorithm include Zhang *et al*.’s [23] dynamic iteration for increased efficiency and Singh *et al*.’s [27] variant for identifying influential community nodes. Additionally, Seifikar *et al*. [28] developed a dynamic version to track evolving social network communities.

The Louvain algorithm’s scalability and effectiveness make it particularly suitable for analyzing alliances in interbank lending networks. Applying this method enables the identification of underlying cooperative structures that shape interbank lending behavior and influence risk contagion within the network.

## Proposed method

### Construction of the initial interbank lending network

The confidentiality of interbank transactions often restricts access to real-world interbank lending data. To overcome this challenge, the Maximum Entropy (ME) method has become a widely used approach for constructing interbank networks. Initially proposed by Jaynes [29] in the study of information theory and probability, the ME model aims to estimate the most unbiased distribution of relationships based on limited known constraints.

In the financial context, banks typically diversify their lending portfolios to maximize returns while minimizing risk. This behavior naturally aligns with the entropy-maximizing principle, making the ME method a robust tool for approximating interbank networks [19,30]. By leveraging this approach, researchers can infer interbank relationships and gain insights into the network’s structure and dynamics, even in the absence of detailed transaction data.

Interbank lending networks often display a characteristic ’core-periphery’ structure, comprising a small, densely connected core of banks and a larger periphery mainly linked to the core instead of to one another. Mathematically, this network is represented by an N×N adjacency matrix, where *N* denotes the total number of banks. Each element *x*_*ij*_ in the matrix quantifies the interbank liability of bank *i* to bank *j*, indicating the amount borrowed by bank *i* from bank *j*. The total interbank assets *A*_*i*_ and liabilities *L*_*i*_ for each bank can be calculated from their balance sheets, using the specific entries under “interbank lending" and “interbank borrowing." This framework provides a comprehensive representation of interbank relationships, facilitating the analysis of systemic dynamics and financial stability.

$$X=\left[\begin{array}{ccccc} x_{11} & \cdots & x_{1j} & \cdots & x_{1N}\\ \vdots & \ddots & \vdots & \vdots & \vdots \\ x_{i1} & \cdots & x_{ij} & \cdots & x_{iN}\\ \vdots & \vdots & \vdots & \ddots & \vdots \\ x_{N1} & \cdots & x_{Nj} & \cdots & x_{NN} \end{array}\right].$$ (1)

Using this data, the interbank lending matrix *X* is derived by solving a constrained optimization problem based on the ME framework. The maximum entropy principle assumes that transaction participants distribute their transaction amounts as uniformly as possible, thereby maximizing the system’s entropy. The optimization problem can be expressed as follows:

$$\max_{x}-\sum_{i=1}^{N}\sum_{j=1}^{N}x_{ij}ln\left(\frac{x_{ij}}{IB_{ij}}\right),$$ (2)

where $IB_{ij}=L_{i}\cdot A_{j}$. The constraints are defined as follows.

$$\sum_{j=1}^{N}x_{ij}=L_{i},$$ (3)

$$\sum_{i=1}^{N}x_{ij}=A_{j},$$ (4)

$$\left\{ \begin{array}{c} x_{ij}\geq 0\;i\neq j\\ 0\;\text{otherwise} \end{array}\right.$$ (5)

Here, *IB*_*ij*_ represents the expected lending volume between bank *i* and bank *j*, calculated as the product of the borrowing capacity *L*_*i*_ of bank *i* and the lending capacity *A*_*j*_ of bank *j*. The RAS algorithm is employed to iteratively solve this optimization problem and find the optimal interbank lending matrix *X*.

The resulting matrix *X* reflects the most probable distribution of interbank lending relationships under the maximum entropy assumption. It effectively captures the diversification strategy pursued by banks, ensuring a balance between risk mitigation and profit maximization within the network.

### Bank alliance detection based on the Louvain algorithm

Interbank networks exhibit intricate and dynamic interactions, particularly in large-scale systems. Identifying cooperative structures within these networks is crucial for understanding systemic dynamics. The Louvain algorithm, known for its computational efficiency and high accuracy in community detection, is well-suited for analyzing large-scale networks. In this study, the Louvain algorithm serves as the core method for uncovering alliance structures within interbank networks. By leveraging its capabilities, we aim to systematically identify potential alliances among banks, providing insights into the underlying cooperative dynamics that shape interbank behavior.

#### Modularity function in the Louvain algorithm.

The Louvain algorithm partitions network nodes into distinct communities by maximizing the modularity function *Q*, which evaluates the quality of the network partition. The modularity function is given by:

$$Q=\frac{1}{2m}\sum_{ij}\left(A_{ij}-\frac{k_{i}k_{j}}{2m}\right)\delta \left(C_{i},C_{j}\right),$$ (6)

$$\delta \left(C_{i},C_{j}\right)=\left\{ \begin{array}{c} 1,\;C_{i}=C_{j}\\ 0,\text{otherwise} \end{array}\right.$$ (7)

where *A*_*ij*_ represents the adjacency matrix of the network. If there is a link between node *i* and node *j*, then *A*_*ij*_ = 1; otherwise, *A*_*ij*_ = 0. *k*_*i*_ and *k*_*j*_ denote the degrees of nodes *i* and *j*, respectively, indicating the total number of connections each node has in the network. *m* represents the total number of edges in the network. $\delta (C_{i},C_{j})$ is the Kronecker delta function, which equals 1 if nodes *i* and *j* belong to the same community ($C_{i}=C_{j}$), and 0 otherwise.

The modularity value *Q* measures the strength of the community structure, with higher values indicating better partitions. A modularity value below 1 suggests that the network is not well-partitioned, while a value close to 0 implies that each node forms its own independent community.

### Workflow of the Louvain algorithm

The Louvain algorithm refines community structures iteratively to maximize the modularity function. Its workflow consists of the following key steps:

(1) Initialization: Each node is initially assigned to its own community, resulting in as many communities as there are nodes.
(2) Local Optimization: For each node, the algorithm calculates the modularity change if the node were moved to the community of one of its neighbors. If the modularity gain is positive, the node is reassigned to the neighboring community that yields the highest increase.
(3) Community Aggregation: Upon completing local optimization, the network is reconstructed by treating each identified community as a “super-node." Edges between these super-nodes are weighted based on the connections between the original nodes, and the process is repeated on the aggregated network.
(4) Termination: The algorithm concludes when no further modularity improvement is achievable, yielding a stable community structure.

Although the Louvain algorithm is computationally efficient for small to medium-sized networks, its scalability is limited in large-scale networks due to the increasing computational complexity. As the number of nodes and edges grows, the algorithm becomes slower and struggles to converge to an optimal solution. Additionally, its reliance on local optimization may result in suboptimal community partitions, particularly in networks with complex structures.

To overcome these challenges, we propose integrating the Louvain algorithm with a heuristic genetic algorithm to enhance alliance detection in large-scale interbank networks. This hybrid approach leverages the Louvain algorithm’s local optimization efficiency while incorporating the global search capabilities of genetic algorithms. The proposed method ensures robust and scalable community detection by exploring a broader solution space and mitigating the limitations of local optimization. The genetic algorithm operates as follows:

(1) Initialization: A population of candidate community structures is randomly generated, with each node assigned to a community. Optionally, the Louvain algorithm’s output can serve as an initial population, providing a strong starting point. The population size *P* is set to 50, and the number of communities is constrained within a range of 5 to 15.
(2) Fitness Evaluation: Each candidate solution is evaluated using the modularity function *Q*, where higher modularity values correspond to better solutions.
(3) Selection and Crossover: High-performing candidates are selected using roulette wheel selection. Offspring are generated by applying a single-point crossover, combining features from parent solutions to create new candidates.
(4) Mutation: To maintain diversity and avoid convergence to local optima, random mutations are applied by reassigning the community memberships of selected nodes.
(5) Convergence: The process iterates for a maximum of 50 generations or until modularity improvements become negligible. The best-performing solution is returned as the final community partition.

By combining the local optimization efficiency of the Louvain algorithm with the global search potential of genetic algorithms, this hybrid framework enhances both the scalability and robustness of alliance detection in large-scale interbank networks. The detailed workflow of the proposed heuristic genetic algorithm is outlined in Algorithm 1.

It is important to note that there are five major commercial banks in China (ICBC, ABC, BOC, CCB, BCOM), meaning that there are at least five central banks. Therefore, the minimum number of alliances is set to 5. During the experiments, it was observed that the number of bank alliances remained below 15 as the number of iterations increased. Consequently, the maximum number of alliances is set to 15 to control the convergence of the genetic algorithm. This parameter can be adjusted based on specific needs, either by considering the number of iterations or by setting a sufficiently large number of alliances as a convergence criterion.

**Algorithm 1. Genetic algorithm for bank alliance partitioning.**



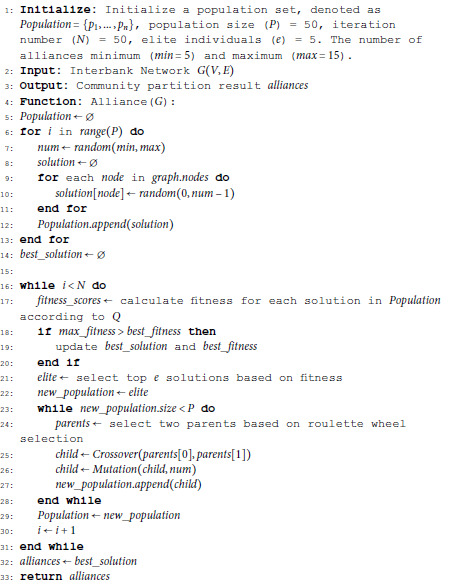



### The alliance-based interbank lend-borrow relationship matching (ABLBM)

In the real-world interbank lending market, borrowing banks exhibit a preference for lenders with whom they maintain established agreements or alliances. They also favor lending institutions with larger asset bases, enabling them to fulfill borrowing needs efficiently while minimizing costs by securing lower interest rates. On the other hand, lending banks prioritize extending credit to allied institutions and favor borrowers with higher creditworthiness, which signals a reduced likelihood of default.

To accurately capture these preferences, we propose a scoring mechanism designed to optimize the matching process between borrowing and lending banks. This mechanism evaluates key factors such as existing alliances, asset sizes, interest rates, and credit risk profiles, ensuring that the modeled interactions closely align with real-world decision-making in the interbank lending market.

#### Borrowing bank scoring mechanism for lending banks.

Let the set of borrowing banks be $br=\{br_{1},br_{2},\ldots ,br_{m}\}$, and the interbank interest rate matrix be represented as:

$$R=\left(\begin{array}{ccc} r_{11} & \cdots & r_{1m}\\ \vdots & \ddots & \vdots \\ r_{m1} & \cdots & r_{mn} \end{array}\right),$$ (8)

where *r*_*ij*_ denotes the interest rate offered by lending bank *j* to borrowing bank *i*. For each borrowing bank *br*_*i*_, the scoring mechanism evaluates lending banks based on their assets, interest rates, and supply capacity. The asset scores are defined as:

$$S_{i,asset}=\frac{Sort_{j,asset}+b}{m+n}\times c,$$ (9)

where ${\text{Sort}}_{j,\text{asset}}$ is the rank of lending bank *j* based on its asset size for bank *j*. *b* and *c* are constants for normalization. The total scores are defined as:

$$S_{i,total}=\alpha *S_{i,asset}+\beta *S_{i,r}+\gamma *S_{i,amount},$$ (10)

where *S*_*i*,*r*_ represents the score based on the interest rate rank. *S*_*i*,*amount*_ denotes the score based on the supply capacity rank. The coefficients α, β, and γ are weight parameters assigned to asset size, interest rate, and supply capacity, respectively. A higher *S*_*i*,*total*_ indicates that the lending bank has a higher priority to be selected by the borrowing bank.

*Remark*: Value for bank asset sizes, interest rates, and creditworthiness scores originate from the CSMAR database’s real data. Creditworthiness scores are calculated by normalizing ranked Moody’s ratings. The parameters α, β, and γ are set to 1 to equally weight the three score components. Note that these weights can be modified by readers for specific applications or simulations. Parameters *b* and *c* are also set to 1, ensuring the score remains within the [0, 1].

#### Lending bank scoring mechanism for borrowing banks.

Let the set of lending banks be $B=b_{1},b_{2},\ldots ,b_{n}$. For each lending bank *b*_*j*_, the scoring mechanism evaluates borrowing banks based on three key factors: assets, creditworthiness, and demand. The total score is defined as:

$$S_{j,total}=\alpha *S_{j,asset}+\beta *S_{j,c}+\gamma *S_{j,amount},$$ (11)

where *S*_*j*,*asset*_ represents the score derived from the rank of the borrower’s asset size, *S*_*j*,*c*_ denotes the creditworthiness score based on rankings of credit ratings (e.g., Moody’s ratings), and *S*_*j*,*amount*_ reflects the rank-based score for the borrower’s demand. The parameters α, β, and γ serve as weight coefficients assigned to asset size, creditworthiness, and demand, respectively. A higher *S*_*j*,*total*_ indicates a stronger preference for the borrowing bank to be selected by the lending bank.

#### Alliance-first matching algorithm.

**Fig 1:**
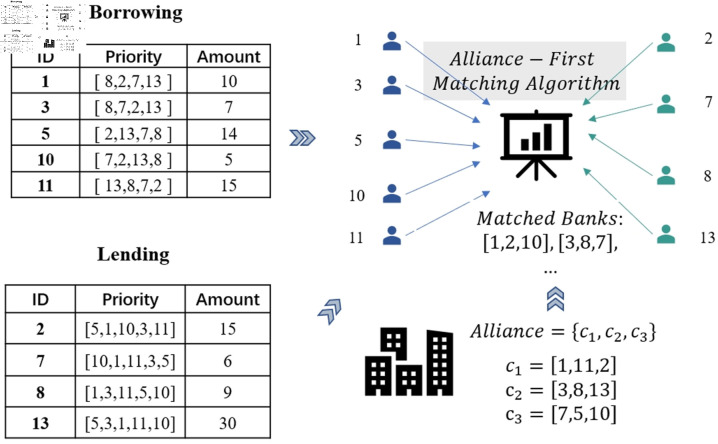
The process of matching borrowing and lending banks using the alliance-first matching algorithm.

The proposed algorithm prioritizes matches within existing alliances. If alliance-based matches cannot fully satisfy the borrowing or lending demands, the algorithm extends to matches outside alliances, prioritizing based on total scores. The matching evaluation function is defined as:

$$M_{\left(i,j\right)}=-\left(\frac{{\mathrm{rank}}_{i,j}}{m}+\frac{{\mathrm{rank}}_{j,i}}{n}\right),$$ (12)

where *ranki*, *j* represents the rank of lending bank *j* as perceived by borrowing bank *i*, *rankj*, *i* is the rank of borrowing bank *i* as perceived by lending bank *j*, *m* denotes the total number of borrowing banks, and *n* represents the total number of lending banks. For example, assume *m* = 5 and *n* = 4. As shown in Fig 1, if borrowing bank 1 prioritizes lending banks as [8, 2, 7, 13] and lending bank 2 prioritizes borrowing banks as [5,1,10,3,11], the matching score *M*_1,2_ is calculated as $M_{1,2}=-\left(\frac{2}{4}+\frac{2}{5}\right)=-0.9$. This scoring function reflects the mutual preferences of borrowing and lending banks, ensuring that the matching process dynamically optimizes the relationship based on both parties’ priorities. By integrating alliance preferences and mutual rankings, the proposed method effectively balances alliance-based matches and external opportunities, ensuring an efficient allocation of lending and borrowing resources in interbank networks.

## Experiments

### Data

This study utilizes data sourced from the CSMAR database (https://data.csmar.com), which provides extensive financial information on Chinese banks, including balance sheet metrics and credit ratings. The balance sheet data encompasses critical financial indicators such as total assets, total equity, total liabilities, interbank assets, and interbank liabilities, enabling a comprehensive assessment of banks’ financial stability and operational performance. Credit ratings are sourced from Moody’s, a globally recognized agency, offering a reliable evaluation of banks’ credit risks and market credibility. These ratings form a vital basis for analyzing interbank lending and borrowing dynamics.

To simulate interbank lending rates, the study leverages market data from the Shanghai Interbank Offered Rate (SHIBOR) (https://www.shibor.org/shibor), which shows that rates for banks of different sizes typically fluctuate between 1% and 3%. This range reflects realistic market conditions and captures the randomness associated with interbank lending. Therefore, we selected the range U(1%,3%) to represent the dynamics of the Chinese interbank market.

**Fig 2:**
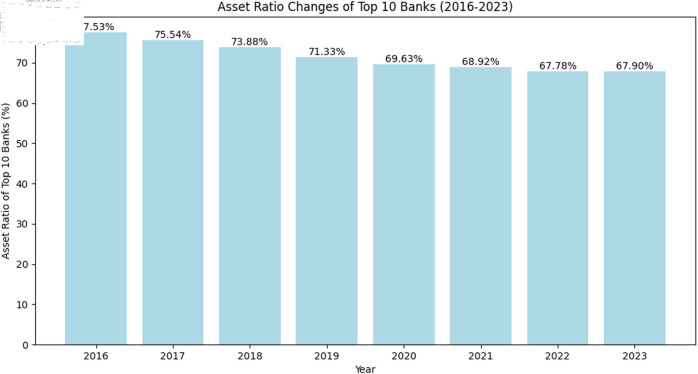
Changes in asset ratios of the top 10 banks from 2016 to 2023.

To make the data structure of this article clearer, we have analyzed the centrality of the Chinese interbank market. According to Fig 2, the asset ratios of the top ten banks have shown a steady decline from 77.53% in 2016 to 67.90% in 2023. This indicates that large-scale banks still hold a significant market share in China’s banking industry, but the market concentration has slightly decreased, suggesting that the market share of smaller banks is gradually increasing.

### Bank alliance partitioning

**Fig 3:**
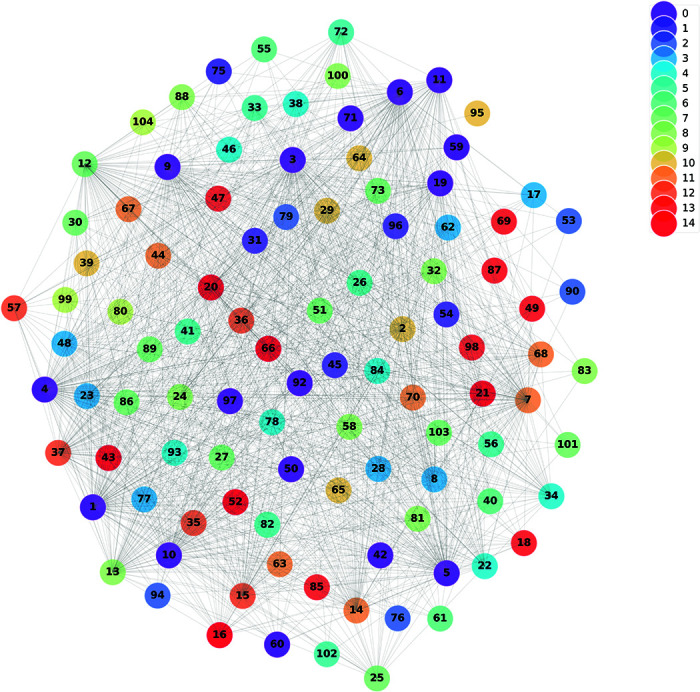
Bank

Using 2023 balance sheet data from Chinese banks, this study constructs an interbank lending network comprising 104 banks. The network models the interactions of interbank lending and borrowing, inferred from the financial data. To uncover the network’s community structure, the Louvain algorithm is employed, effectively partitioning the network into distinct alliances. The results are visualized in Fig 3, with each color indicating a specific alliance. Nodes represent individual banks, uniquely labeled by numerical identifiers (IDs) for clarity, while edges denote interbank lending relationships.

The visualization highlights both the alliances formed among banks and their underlying transactional connections. This community structure offers valuable insights into interbank lending preferences and the role of alliances in influencing network stability and systemic risk. Subsequent analysis examines the impact of these alliances on the dynamics of interbank borrowing and lending, as well as their effectiveness in mitigating financial risks and enhancing network resilience.

### Simulation experiments

#### Simulation setup.

**Fig 4:**
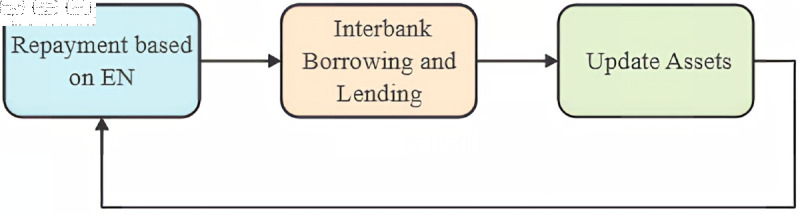
Simulation process.

**Fig 5:**
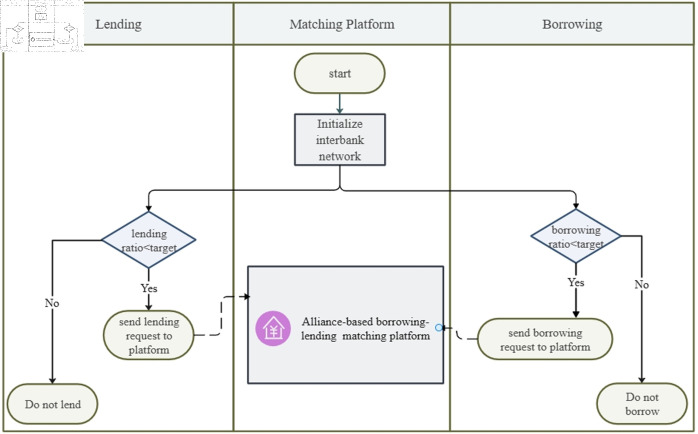
Lending and borrowing process.

To study the dynamics of interbank networks, this research employs an agent-based modeling (ABM) approach. The simulation is based on annual balance sheet data from 2016 to 2023, summarized in Table 1. The simulation period is set to one year and encompasses processes such as repayment, lending and borrowing, and asset updates, as illustrated in Fig 4 and detailed in Fig 5.

**Table 1:** Balance sheet items.

Assets: A	Liabilities: L
Overnight lending: ON	Overnight borrowing: ON
Short-term lending: ST	Short-term borrowing: ST
Long-term lending: LT	Long-term borrowing: LT
Cash and balance due	Other liabilities
Other assets	Equity: E

**Fig 6:**
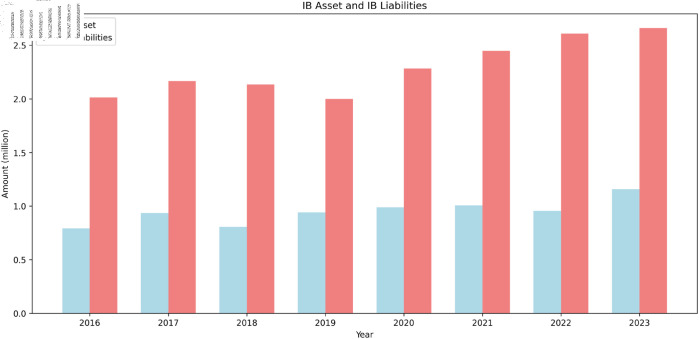
Annual total interbank borrowing assets and liabilities.

During the lending and borrowing process, each bank identifies its borrowing and lending counterparts based on its borrowing-to-lending ratio, as outlined in Table 2. Interbank transaction data is generated using the banks’ annual borrowing and lending totals, as illustrated in Fig 6. On average, interbank assets account for approximately half of a bank’s liabilities. To capture this characteristic, target lending demands are set to 50% of the actual interbank asset values, ensuring balanced capital flows throughout the network.

**Table 2:** Borrowing categories and targets.

Borrowing/lending Type and Ratio	Borrowing/lending Target
Overnight lending, borrowing ratio	(ON/A), (ON/L)
Short-term lending, borrowing ratio	(ST/A), (ST/L)
Long-term lending, borrowing ratio	(LT/A), (LT/L)

Banks with unmet borrowing or lending needs submit requests to the centralized matching platform. This platform simulates the core function of the real-world interbank market, facilitating transactions where commercial banks seek counterparties for liquidity, with allocation driven by our algorithm. It should be noted that this platform is distinct from and does not mimic the role of the central bank. The platform allocates resources using an alliance-based matching algorithm. This approach ensures that both borrowing and lending preferences are optimally addressed. Following each transaction, the financial states of all participating banks are updated, reflecting their revised asset and liability conditions, thereby maintaining an accurate and dynamic representation of the network’s financial landscape.

#### Impact of alliance-based network structure on risk contagion.

This study evaluates the impact of alliance-based network structures on risk contagion by comparing three network generation methods: the Maximum Entropy (ME) method, the Minimum Density (MD) method [31], and the proposed Alliance-Based Lend-Borrow Matching (ABLBM) method. The analysis focuses on structural characteristics such as clustering and information propagation to assess their influence on risk contagion. The following metrics are used for evaluation:

- **Density** measures the ratio of actual edges to possible edges in the network. A higher density indicates a more interconnected network, facilitating information flow and risk propagation.
- **Clustering Coefficient** quantifies the tendency of nodes to form tightly connected groups, reflecting the connectivity of a node’s neighbors.
- **In-Clustering** represents the extent to which a node belongs to a specific cluster, describing its affiliation within the community structure.
- **Out-Clustering** measures a node’s connection to other clusters, reflecting its relationships beyond its immediate community.
- **Power Law** describes the statistical distribution of connections in the network, commonly observed in interbank networks.
- **Average Betweenness** evaluates node centrality by indicating the proportion of shortest paths passing through a node. Nodes with higher betweenness play a critical intermediary role within the network.

The performance of these methods is detailed in Table 3. The Maximum Entropy (ME) method generates the densest network with the highest clustering coefficient, indicating a high level of structural cohesion and efficient information flow. In contrast, the Minimum Distance (MD) method results in a significantly sparser network with weaker clustering, which may hinder effective information transmission.

**Table 3:** Performance of different network generation methods on network metrics.

Network	Density	Clustering Coef.	In-Clustering	Out-Clustering	Power Law	Avg Betweenness
ME	0.1099	0.8217	0.6992	0.7591	0.3027	0.0065
MD	0.0178	0.0903	0.0023	0.0059	0.4667	0.0285
ABLBM	0.0283	0.2259	0.1104	0.0639	0.5625	0.0260

Our ABLBM method achieves notable improvements in clustering and connectivity, particularly within alliances, enhancing both information propagation and overall network resilience. Nodes in ABLBM-generated networks exhibit pronounced in-degree and out-degree characteristics, highlighting the pivotal roles played by certain banks in facilitating interbank interactions. This community-driven structure fosters the development of stable subnetworks, offering enhanced efficiency and robustness when compared to the ME and MD methods.

To evaluate risk contagion within interbank networks, the DebtRank model [32] is employed as a quantitative framework for measuring both individual bank losses and the systemic risk of the entire network. DebtRank captures the propagation of risk by quantifying the relative equity losses incurred by banks during financial distress. The relative loss for an individual bank *i* at time *t* is defined as:

$$h_{i}(t)=\frac{E_{i}(0)-E_{i}(t)}{E_{i}(0)},$$ (13)

where *h*_*i*_(*t*) denotes the relative loss of bank *i*, *E*_*i*_(0) is its initial equity, and *E*_*i*_(*t*) represents its equity at time *t*.

Furthermore, the iterative formula for the relative loss of bank *i* during the contagion process is expressed as:

$$h_{i}(t+1)=h_{i}(t)+\sum_{j=1}^{N}\Lambda_{ij}\left[h_{j}(t)-h_{j}(t-1)\right],$$ (14)

where $\Lambda_{ij}=\frac{IB_{ij}}{E_{j}}$ represents the contagion coefficient, which quantifies the impact of borrowing bank *j* on lending bank *i*. Here, *IB*_*ij*_ denotes the interbank lending volume between banks *i* and *j*, while *E*_*j*_ refers to the equity of bank *j*. This iterative formula reflects how the relative equity loss of bank *j* propagates through the network, affecting other connected banks over time.

The systemic risk of the entire network at time *t*, denoted as *H*(*t*), is calculated as:

$$H(t)=\sum_{i=1}^{N}\left(h_{i}(t)\frac{E_{i}(0)}{\sum_{j=1}^{N}E_{j}(0)}\right),$$ (15)

where *N* represents the total number of banks in the network. *H*(*t*) provides a comprehensive measure of the aggregate risk, considering both the relative losses of individual banks and their initial equities. The DebtRank model, through its quantification of both localized and systemic risk, offers valuable insights into the dynamics of risk contagion. It accounts for the interconnected nature of interbank networks and the cascading effects of financial stress, thereby providing a robust framework for assessing systemic vulnerabilities.

The simulation in this article involves 104 large, medium, and small banks in China. To simulate external shocks, this study imposes a 10%–100% asset loss on individual banks to model the impact of financial crises.

**Fig 7:**
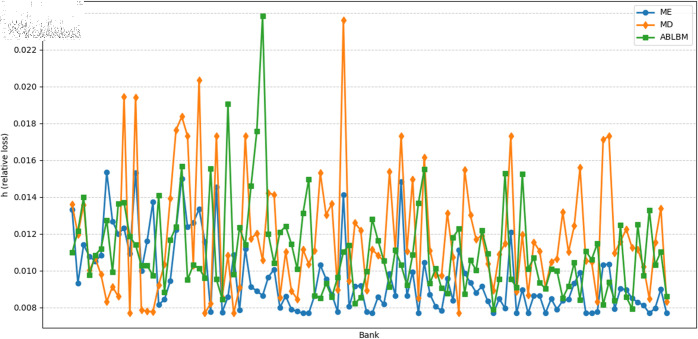
Relative loss h for each bank under a large-scale shock.

The simulation results exhibit different characteristics depending on the scale of the shock. Under large-scale shocks (Impact each bank sequentially, with an impact size of 80%), results as shown in Fig 7, reveal that the ABLBM network demonstrates moderate clustering and stability, with systemic losses comparable to other methods (ME and MD). This suggests that the alliance-based structure does not exhibit significant vulnerability during systemic crises.

**Fig 8:**
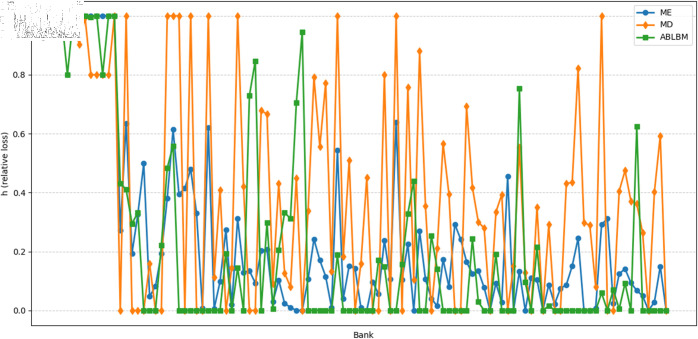
Relative loss h for each bank under a small-scale shock.

**Fig 9:**
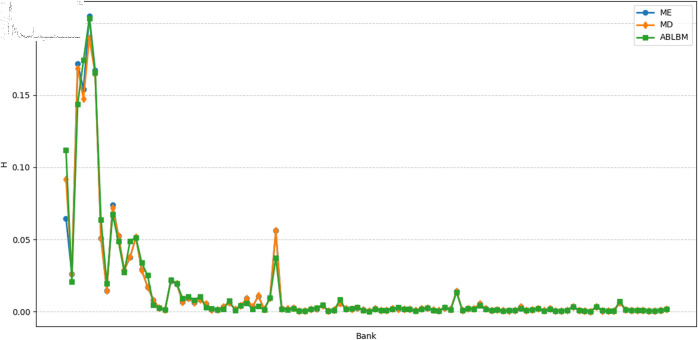
Total relative loss of the banking system under each shock.

Under small-scale shocks shown in Fig 8 (The simultaneous shock on 10 banks is 80%), the ABLBM method exhibits a distinct pattern of risk contagion. We observed relatively weaker inter-alliance contagion and stronger intra-alliance contagion, which could facilitate better risk absorption within alliances. Systemic risk as shown in Fig 9, and the three exhibited similar volatility.

To more clearly quantify the impact of alliance structures on risk during contagion, we recorded the means and variances of bank losses for ME, ABLBM, and MD, as shown in Table 4, the ME method performs best according to both metrics, showing the lowest average loss and the lowest variance (highest stability). The MD method performs highest on both metrics. The ABLBM method demonstrates an intermediate performance, achieving lower average losses and less variance compared to the MD method. Overall, these findings suggest that ABLBM demonstrates resilience under different shocks.

**Table 4:** Statistical table of relative loss (h).

Method	Sequential Shocks (100%)		Sequential Shocks (80%)		Simultaneous Shocks (10 banks for 80%)	
	Mean	Variance	Mean	Variance	Mean	Variance
ME	0.011992	0.009596	0.009593	0.006141	0.237117	0.084558
MD	0.014488	0.012611	0.011803	0.008447	0.384093	0.131642
ABLBM	0.013793	0.011320	0.011073	0.007312	0.204439	0.103035

Based on the findings, we recommend that banks focus on strengthening or forming interbank alliances to enhance interbank connectivity. Our analysis, utilizing the ABLBM structure which reflects real-world connectivity levels, alongside simulations showing greater resilience in densely connected networks (such as the maximum entropy model under our scenarios), indicates that fostering these interbank connections is crucial for improving their ability to manage risks, particularly when facing / during shocks. Real-time monitoring of asset losses and exposures is essential for identifying and addressing potential risks promptly, minimizing their impact on financial stability. For regulatory authorities, fostering transparency and effective communication among banks is crucial for sharing best practices and enhancing systemic resilience. Governments should consider introducing policy incentives to encourage the formation of stronger interbank alliances, promoting collaboration and reinforcing the stability of the financial system. By supporting these initiatives, both banks and regulators can contribute to a more robust and interconnected banking network capable of withstanding systemic pressures.

**Fig 10:**
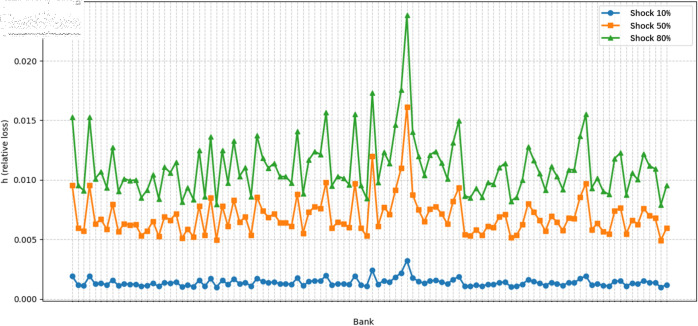
The impact of different attack rates on the relative loss h of bank IDs.

To verify the sensitivity of the system under different levels of impact, we conducted tests on 10 banks with shocks of 10%, 50%, and 80%, demonstrating the effects of different attack rates on the relative losses of these banks. As the attack rate increased from 10% to 80%, as shown in the Fig 10, the relative losses rose significantly, particularly under high shock conditions, with some banks exhibiting clear vulnerability and spikes in losses, indicating a higher sensitivity to shocks.

## Conclusion

This study explores alliance-based interbank lending networks, highlighting the pivotal role of alliances in mitigating risk contagion. Departing from traditional approaches that predominantly focus on historical relationships and credit factors, this research addresses the complexity of long-term cooperative relationships by introducing the ABLBM to capture alliance-driven interbank lending behaviors. Simulation results demonstrate that alliance-based networks (compared to other network structures like the MD model under our scenarios) exhibit greater resilience. This indicates that fostering and strengthening interbank alliances is crucial for improving the banking system’s ability to manage risk, particularly when facing shocks. Real-time monitoring of asset losses and exposures is essential to promptly identify and address potential risks, minimizing their impact on financial stability. For regulatory authorities, fostering transparency and effective communication among banks, particularly concerning information sharing on alliance structures and risks, is crucial for promoting best practices and enhancing systemic resilience. Governments should consider introducing policy incentives to encourage the formation of stronger interbank alliances, thereby promoting collaboration and reinforcing the stability of the financial system. By collaboratively advancing these initiatives, banks and regulators can contribute to building a more robust and interconnected banking network capable of withstanding systemic pressures.
